# Development and Psychometric Properties of Menstrual Health Seeking Behaviors Questionnaire (MHSBQ-42) in Female Adolescents

**Published:** 2018

**Authors:** Fatemeh Darabi, Mehdi Yaseri, Alireza Rohban, Farideh Khalajabadi-Farahani

**Affiliations:** 1- Department of Public Health, Asadabad School of Medical Sciences, Asadabad, Iran; 2- Department of Epidemiology and Biostatistics, Tehran University of Medical Sciences, Tehran, Iran; 3- Department of Rehabilitation Management, School of Rehabilitation Sciences, Iran University of Medical Sciences, Tehran, Iran; 4- Department of Population & Health, National Population Studies & Comprehensive Management Institute, Tehran, Iran; 5- Department of Midwifery, Sexual & Reproductive health Research Centre, Mazandaran University of Medical Science, Sari, Iran

**Keywords:** Female adolescents, Menstrual health, Psychometric, Questionnaire, Tehran

## Abstract

**Background:**

Lack of accurate menstrual knowledge, attitude, and practices leave female adolescents ignorant of the necessary health behaviors during menstruation. This study aimed to develop a menstrual health-seeking behavior questionnaire based on the theory of planned behavior to evaluate its psychometric properties in female adolescents in Tehran.

**Methods:**

This study was conducted on 578 female adolescents aged 12–15 years in Tehran. The first draft of the menstrual health-seeking behavior questionnaire containing 52 items was developed based on the theory of planned behavior (TPB). The content and face validity of the questionnaire was assessed by a panel of experts. Construct validity was also assessed using exploratory factor analysis (KMO=0.73) with varimax rotation. Cranach’s alpha and test-retest were used to examine the reliability of the questionnaire. All statistical analysis was performed using SPSS 23.0 and AMOS 23.0.

**Results:**

The content and face validity of the 42 items were finally confirmed. Content validity index was greater than 0.73 for all six TPB constructs. Explanatory factor analysis yielded an acceptable fit for the six-factor model (RMSE=0.053, 95% CI 0.042–0.064). These factors jointly explained 65% of the variance in the outcome variables. Cranach’s alpha coefficients ranged from 0.79 to 0.91, demonstrating an excellent internal consistency and high reliability of the questionnaire. Test-retest reliability was also satisfactory for all items (ICC=0.86–0.94).

**Conclusion:**

The results illustrate that the menstrual health-seeking behavior questionnaire is psychometrically adequate and highly reliable. This theoretically grounded questionnaire can be well applied in future interventions for female adolescents to assess their menstrual health-related knowledge, attitude, and practices.

## Introduction

Puberty is a stage of rapid physical changes in a child’s body through which the child becomes an adult capable of reproduction. Menarche is a unique marker of maturation in girls (1) that occurs at the mean age of 12.8 years according to a systematic review in Iran (2). Studies in developed countries have reported that most adolescents (65%) have insufficient knowledge about puberty and sexuality (3). In addition, the lower knowledge has been observed in adolescent girls (4). Little access to reliable information about reproductive health (5), lack of appropriate mother-daughter communication regarding menstruation (6), socio-cultural restrictions (7), and taboos surrounding menstruation (8) lead to lack of necessary knowledge, attitude, and practices regarding menstrual health among adolescent girls. Accurate menstrual health knowledge and practices not only prevent reproductive tract infections (7), but reduce school absentees, abnormal abdominal pain, scabies in the vaginal area, and other complications (7, 9, 10). Iranian adolescents comprise a significant proportion (14.3%) of the whole country’s population. According to 2016 Iranian population and housing census, there were approximately 11 million adolescents aged 10–19 across the country (11). Despite the high number of Iranian adolescents and the importance of menstrual health issues for adolescent girls, few studies in Iran have been developed and few questionnaires for assessing puberty and menstrual health-seeking behavior among female adolescents were evaluated (3, 12). Rabiepoor et al. (2017) (13) and Alizadeh et al. (2011) (14) have developed a questionnaire which assesses only puberty and menstrual health knowledge among Iranian female students. Afghari et al. (2008) have developed a self-made questionnaire to assess the knowledge and attitude of female adolescents regarding puberty-related issues in Isfahan province, Iran, but they have not evaluated the questionnaire in terms of reliability and validity (12). Having a validated and culturally-tailored questionnaire to assess menstrual health**-**seeking behavior is a prerequisite for the effective educational and behavioral interventions among female adolescents.

Theory of Planned Behavior (TPB) is one of the commonly used and reliable behavior change theories (15) and probably the most influential theory to predict social and health behaviors among adolescents (16). Several recent studies have emphasized the effectiveness of TPB-based interventions for reducing high-risk sexual behaviors and improving physical activity, nutritional behaviors, and reproductive health behaviors among female adolescents (17–19). Based on TPB, there are three determinants of behavior which include attitudes, subjective norms, and perceived behavioral control. Some studies have shown that the perceived parental control may improve children’s behaviors as well. Thus, in this study, the construct of perceived parental control was also added to the conceptual model of the study to strengthen the predictive power of the questionnaire. Finally, the menstrual health**-**seeking behavior questionnaire was developed based on the constructs of TPB and its reliability and validity were evaluated with the help of psychology and health experts using statistical analysis.

## Methods

### Questionnaire development:

First of all, some items from the WHO questionnaire were extracted, which include items for assessing knowledge of reproductive health and its sources as well as sexual attitudes and behaviors (20). Then, electronic databases were investigated to find key concepts associated with adolescent menstrual and reproductive health. Of the total 317 papers found, 71 were considered eligible for careful review. Among them, 15 papers were identified to be closely relevant to adolescent menstrual and reproductive health. After that, the study purpose and method, the data gathering tool, target group, and methods used for evaluating study variables for each of these 15 papers were all determined. Next, five papers were selected and relevant items were extracted. In addition, eight one-hour Focus Group Discussions (FGDs) were run with 40 participants to explore for some other relevant items. Finally, some items were added and some were removed from the final draft of questionnaire.

### Demographic and knowledge items:

The demographic variables included age, father’s and mother’s education/job, and self-reported economic situation. The adolescent menstrual health knowledge was assessed using 10 items. Responses were scored using three categories; true, false, and do not know. Every correct answer was given a score one and incorrect and unknown answers were given a score zero.

### TPB constructs:

The questionnaire containing 51 items in six parts was developed to measure TPB constructs in relation to menstrual health. Nine items were used to assess the adolescents’ perception of behavioral control on how to behave to maintain their menstrual health. The influence of significant others (Family, friends, and teachers) on adolescents’ opinion about menstrual health was assessed by 10 items. In addition, the adolescent menstrual health attitude was assessed using 11 items. Six items were used to assess the behavioral intention towards menstrual health practices. Eight items were used to assess menstrual health practices among female adolescents. Furthermore, seven items were used to assess perceived parental control. All items were scored on a five-point Likert scale, ranging from “Strongly disagree” to “Strongly agree”. Final scores were expressed as 0 to 100 values using the following formula in order to make the results comparable with the other studies.

$$\mathrm{New}\;\mathrm{score}\;=\;100\times \frac{\mathrm{Score}-\mathrm{minimum}\;\mathrm{possible}\;\mathrm{score}}{\mathrm{range}\;\mathrm{of}\;\mathrm{scores}}$$

The minimum possible attainable score for each item was one and the range of scores was four (5-1). The total scores for each construct were calculated by averaging the scores of all items on that construct. Each participant could obtain the maximum of 100 scores for each construct and the questionnaire overall. The total scores were categorized into three classes; poor (0 to 33.3), moderate (33.4 to 66.7), and good (66.8 to 100).

### Psychometric properties

#### Face validity:

Face validity was assessed through qualitative and quantitative methods. The expert panel consisted of 11 academic members and specialists in the fields of health education and promotion, public health, and psychology, from the schools of public health, and nursing and midwifery reviewed the items to ascertain whether they can appropriately assess what they intended to assess. In addition, using convenience sampling, ten female adolescents were recruited to determine if the items were relevant, understandable, and clear. At the next stage, the impact score was used and 12 female adolescents assessed the importance of each item using five-point Likert scale (Very important=score 5, important=score 4, moderately important=score 3, slightly important=score 2, and not at all important=score 1). Items with mean scores lower than 1.5 were omitted from the questionnaire.

#### Content validity:

Content validity was assessed through qualitative and quantitative methods. For the qualitative assessment, the expert panel assessed the items with respect to the scaling, wording, and item allocation. Then, their corrective comments were applied to the questionnaire. For quantitative assessment, 12 experts, in the fields of health education and promotion, and psychology were asked to assess the necessity of each item on a 3-point Likert scale (1=essential, 2=useful but not essential, and 3=not essential) in order to calculate Content Validity Ratio (CVR). According to Lawshe’s table (21), items with CVR value of 0.54 and above remained in the questionnaire and the rest were omitted. In addition, using the 4-point Likert scale, the simplicity, relevancy, and clarity of all items were assessed by the expert panel in order to calculate Content Validity Index (CVI). According to Waltz and Bausell index (22), items with a CVI value of 0.79 and above remained in the questionnaire and the rest were omitted.

#### Construct validity:

The construct validity was assessed using Exploratory Factor Analysis (EFA). Varimax rotation was employed to examine the factor structure and estimate the factor loading. In addition, KMO and Bartlett’s test of sphericity was used to determine whether the sample is adequate for the factor analysis. Eigenvalues above one and scree plot were computed to determine the number of factors. Factor loadings equal to or greater than 0.4 were considered appropriate (23). Furthermore, Confirmatory Factor Analysis (CFA) was performed to assess the model fitness. Various fit indices such as relative Chi-square and degrees of freedom (*χ*^2^/df), Comparative Fit Index (CFI), Normed Fit Index (NFI), Incremental Fit Index (IFI), Root Mean Square Error of Approximation (RMSEA), and Standardized Root Mean Square Residual (SRMR) were used to determine the fit of the model.

#### Participants:

This methodological and cross-sectional study was conducted among 578 female adolescent students aged 12–15 years in Tehran, the capital of Iran, in 2016.

#### Selection of subjects:

The sample size estimation was based on knowledge as it gives us the maximum sample size. Since there were 52 items for 7 latent variables, 412 samples were needed when the alpha level was considered to be 5%. As a design effect of 1.4 was expected, the sample size was increased to 578 (24). The inclusion criteria were as follows: a female adolescent student aged 12–16, residing in Tehran, and willing to participate in the study.

#### Reliability assessment:

The reliability of the total questionnaire and every construct were assessed by Cranach’s Cronbach’s alpha (α) coefficient. Furthermore, test-retest reliability was assessed using the Intraclass Correlation Coefficient (ICC) for 30 female adolescents with a 2-week interval.

#### Statistical analysis:

All analysis was carried out using SPSS 23.0 and AMOS 23.0 software. Descriptive statistics such as mean, standard deviation, frequency, and percentage were used for the demographic characteristics of the study participants. All statistical tests were two-sided and p≤0.05 was considered to be statistically significant.

## Results

The total number of participants was 578 with a mean age of 14.1±1. Table 1 presents the demographic characteristics of the study participants. Questionnaire validation at different aspects was shown as the following:

**Table 1.** Demographic characteristics of the study participants (n=578)

**Variable**	**Sub-variable**	**Total no. (%)**
**Economic situation**		
	Very good	35 (6.1%)
	Good	242 (41.9%)
	Average	254 (43.9%)
	Weak	47 (8.1%)
**Father’s education (year)**		
	<6	57 (9.9%)
	6–12	366 (63.8%)
	>12	151 (26.3%)
**Mother’s education (year)**		
	<6	72 (12.5%)
	6–12	379 (65.8%)
	>12	125 (21.7%)
**Fathers occupational status**		
	Employed	544 (94.1%)
	Unemployed	34 (5.9%)
**Mother’s occupational status**		
	Employed	153 (26.5%)
	Housewife	425 (73.5%)

### Face validity:

In the qualitative part of the assessment, the expert panel suggested removing two items due to lack of consistency between the items and their equivalent category and conceptual overlap. In the quantitative part of the assessment, one item had an impact score of less than 1.5 and, consequently, was omitted.

### Content validity:

In the qualitative part of the assessment, four items were found to be inappropriate in terms of scaling, wording, and item allocation, and they were omitted from the questionnaire. In the quantitative part of the assessment, one item with CVR less than 0.54 and CVI less than 0.80 was omitted. The mean CVR and the mean CVI were 0.66, and 0.73, respectively.

### Construct validity:

**Figure 1.**
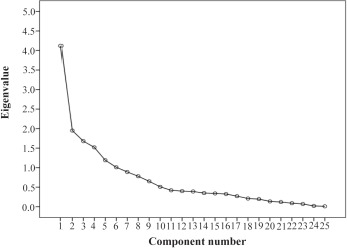
Scree plot

The construct validity was assessed using Exploratory Factor Analysis (EFA). Varimax rotation was employed to examine the factor structure and estimate the factor loading. In addition, KMO and Bartlett’s test of sphericity was used to determine whether the sample is adequate for the factor analysis. Eigenvalues above one and scree plot were computed to determine the number of factors (Figure 1). Factor loadings equal to or greater than 0.4 were considered appropriate Principal component analysis with varimax rotation identified six factors with eigenvalues greater than one and factor loadings equal to or more than 0.4. Taken together, these factors explained 65% of the variance observed. In addition, the KMO was equal to or less than 0.73 and Bartlett’s test reached statistical significance (p< 0.001). The analysis of subscale showed an acceptable level of homogeneity. Two items with factor loadings less than 0.4 were omitted.

### The factor loadings were as follows:

Factor 1 (Perceived behavioral control) including 6 items, Factor 2 (Subjective norms) including 7 items, Factor 3 (Attitudes towards menstrual health issues) including 11 items, Factor 4 (Behavioral intention) including 6 items, Factor 5 (Menstrual health behaviors) including 8 items, Factor 6 (Perceived parental control) including 4 items. In total, the questionnaire included 42 items (Table 2).

**Table 2.** Constructs of the menstrual health-seeking behavior questionnaire for female adolescents (n=578)

**Construct**	**Number of items**	**Mean±SD**	**α (N=578)**	**ICC (N=578)**
**Attitudes towards menstrual health**	11	41.0±9.4	0.85	0.94
**Subjective norms**	7	30.0±20.2	0.89	0.92
**Behavioral intention**	6	31.4±12.8	0.80	0.91
**Perceived parental control**	4	43.1±15.1	0.91	0.92
**Perceived behavioral control**	6	32.2±15.1	0.79	0.93
**Menstrual health behaviors**	8	42.3±16.1	0.89	0.94
**Total**	42	39.6±7.9	0.91	0.86

CFA indicated a good fit of the six-factor model. Further, all indices showed acceptable results (The relative *χ*^2^/df=1.911<3, p<0.001; RMSEA= 0.053>0.08, (95% CI=0.042–0.064); CFI=0.962 >0.9; IFI=0.955>0.9; TLI=0.946>0.9; GFI=0.996 >0.9; AGFI=0.995). Therefore, the final model with 42 items was appropriate for this study population (Table 3).

**Table 3.** Goodness of fit indices for menstrual health-seeking behavior questionnaire

**Construct**	**RMSEA**	**LO_90_ HI90**	***χ*^2^/df**	**TLI**	**IFI**	**NFI**	**CFI**	**AGFI**	**GFI**	**SRMR**
**Summary of rules of thumb**	<0.5^*^		0.00	>0.8			>0.9			<0.6
	<0.1^**^									<4
**Adolescent menstrual health**	0.053	0.042	1.91181	0.946	0.955	0.955	0.962	0.995	0.996	0.056
		0.064								

* Good fit,

** Mediocre fit

### Reliability:

The overall questionnaire and its constructs had excellent internal consistency with Cronbach’s α coefficient of 0.92 for the overall questionnaire, and ranges of 0.79 to 0.91 for the constructs. Test-retest results indicated excellent reliability for all constructs (ICC≥0.80) and the total scores revealed that the menstrual health-seeking behavior questionnaire is an internally consistent and psychometrically sound questionnaire over time (ICC=0.86–0.94).

## Discussion

The results supported the validity and reliability of the questionnaire in the sample of female adolescents. The initial questionnaire consisted of 52 items. However, after rigorous psychometric evaluation, the number of items was reduced to 42. The results of the content and face validity showed that the items were comprehensible and culturally-tailored to the Iranian female adolescents. Content validity was assessed by a team of experts and the results indicated a satisfactory level of validity and reliability. The 42-item questionnaire consisted of six different constructs of TPB to measure different aspects of menstrual health among female adolescents. EFA with varimax rotation showed that six factors (Including attitude, subjective norms, perceived behavioral control, perceived parental control, behavioral intention, and behavior) could be extracted. According to the results obtained by KMO analysis, the sample size was adequate and the factor analysis was favorable. The six-factor model of the menstrual health-seeking behavior questionnaire accounted for 65% of the total variance (Table 4).

**Table 4.** Results from the EFA with varimax rotation in female adolescents aged 12–16 in Tehran (n=578)

**No.**	**Items**	**Factor 1**	**Factor 2**	**Factor 3**	**Factor 4**	**Factor 5**	**Factor 6**
1	I can take a shower during my menstrual period.	0.870to 0.771				
2	It is easy for me to control my mood swings such as depression during menstruation period.					
3	It is easy for me to communicate with others during the menstruation period.					
4	I am sure, I can do more social activities during puberty.					
5	It is easy for me to look after my individual menstrual health such as washing after each bowel movement during the menstrual period.					
6	I am sure I can manage major signs of the puberty.					

7	Important people to me, think that I should take a standing shower during menstruation.		0.875 to 0.665				
8	My family believes that I should continue my social activities during menstruation.						
9	My family believes that I should follow my social activities during my menstrual period similar to before.						
10	Important people to me think that I should follow a proper diet during the period of puberty.						
11	The people around me, want me to look after my personal menstrual health in order to prevent any infection.						
12	Most of my friends agree with the lack of desire for doing homework due to mood swings caused by menstruation.						
13	Important people to me think that physical and emotional changes during puberty prevent me from carrying out social activities such as attending social meetings.						

14	Carrying out the correct cleaning after each bowel movement individual prevent Pelvic Inflammatory Disease.			0.749 to 0.559			
15	Changing underwear daily is quite an important behavior among women to prevent uterine infection.						
16	Taking a standing shower, especially during the menstrual period, is quite an important behavior.						
17	Personal hygiene during menstruation (Such as cleaning the vulva area and the anus area after each excretion and defecation) is essential.						
18	Individual health during menstruation prevents the risk of infection.						
19	Puberty reduces the interest in daily activities.						
20	Oversleeping in the menstrual period leads to boredom for doing homework.						
21	Puberty causes a sharp and aggressive behavior in dealing with others.						
22	Menstruation disturbs the everyday life activities.						
23	Menstruation causes difficulties in concentrating on some activities such as education.						
24	Menstruation decreases the interest in doing school activities.						

25	I have decided to frequently change my menstrual pad during my menstrual period.				0.726 to 0.625		
26	I want to follow health matters during my menstrual period (Such as bathing, changing underwear, etc.).						
27	I have planned to follow health matters during my menstrual period (Such as bathing, changing underwear, etc.).						
28	I have decided to continue my social activities during my menstrual period similar to other ordinary days.						
29	I’m going to wash after each bowel movement during menstruation.						
30	I’m going to follow health matters during the period of puberty (Such as bathing, changing underwear, etc.).						

31	During my menstrual period, I change my underwear every day.					0.743 to 0.619	
32	I take a standing shower, especially during my menstrual period.						
33	I follow the puberty-related health issues, such as menstrual health.						
34	After each bowel movement, I wash the vulva and the anus area properly from front to back to prevent Pelvic Inflammatory Disease.						
35	I don’t go to the sea and the pool during my menstrual period.						
36	I avoid drinking caffeinated drinks before the onset of a menstrual period in order to prevent premenstrual symptoms (e.g., nervousness, menstrual pain, etc.).						
37	I would use cotton underclothes during my menstrual period.						
38	During my menstrual period, I carry out those activities that can reduce aggression.						

39	My parents determine how much I should read about the puberty health-related issues.						0.880 to 0.821
40	My parents provide me with the necessary training and guidance about the puberty health-related issues.						
41	My parents provide me with the necessary training and guidance about the menstrual health-related issues.						
42	My parents determine how much I should read about the menstrual health-related issues.						

43	Eigenvalues	4.12	1.95	1.68	1.52	1.19	1.01

44	Variance explained (%)	22.93	10.83	9.35	8.48	6.61	5.66

45	Cumulative variance (%)	22.93	33.76	43.12	51.61	58.23	65.01

Factors: 1=perceived behavioral control, 2=subjective norms, 3=attitude, 4=behavioral intention, 5=behavior, 6=perceived parental control

The results of CFA in the study of Barati et al. among adolescents showed that the six factors explained 62% of the total variance (15). Another study on youth showed consistent results and the model explained 64% of the total variance (25). Similar to the study conducted by Barati et al., our data showed normative patterns (15). The obtained values of CVI and CVR in our study were reasonable and CVR value of ≥0.54 and CVI value of ≥0.79 was considered acceptable. In contrary, in another study (26), which assessed the reliability and validity of the international AIDS questionnaire for Iranian student, the CVR and CVI values more than 0.7 were considered satisfactory.

Test-retest results indicated excellent reliability for all constructs (ICC≥0.80) and the total scores confirmed the stability and reliability of the menstrual health-seeking behavior questionnaire. Our results are not in line with the results obtained by previous studies (16, 27). Analysis of the item-to-total correlations revealed an identical question pattern similar to what reported by Valizadeh et al. (2017) (28), and Barati et al. (2015) (15). A Cronbach’s α coefficient of 0.92 for the overall questionnaire was obtained in this study that is in agreement with the study (29) which obtained the Cronbach’s alpha coefficient of 0.91.

The development of a theory-driven questionnaire for assessing menstrual health-seeking behavior among female adolescents was the key strength of this study. This questionnaire is a simple, valid, reliable, context-based, and practical tool. Careful selection of items relevant to menstrual health issues might be a good reason for obtaining such satisfactory results. The questionnaire was developed and validated based on Iranian socio-cultural context. However, its validity has not been assessed for other cultures. Therefore, further studies should be conducted to confirm the validity and applicability of the questionnaire for different cultures. As of today, such theory-based menstrual health-seeking behaviors questionnaires had not been developed and assessed for female adolescents in Iran. While the previous questionnaires have been developed to only assess knowledge and attitudes towards puberty and menstrual health issues, this questionnaire benefits from the evaluation of psychometric properties of the behavior without losing any important aspect of the menstrual health-seeking behavior. This questionnaire is short and easy to use.

Despite the strengths mentioned, the present study had a limitation; as the questionnaire was developed for Iranian female adolescents, and due to the cultural diversity, this questionnaire can’t be applied to other female adolescents who belong to different cultures at various geographic locations.

## Conclusion

The menstrual health-seeking behaviors questionnaire is a well-established, psychometrically adequate, and highly reliable questionnaire that can be applied in future interventions on female adolescents to assess menstrual and puberty health-seeking behaviors among them.

## References

[1] McGrory A. Education for the menarche. Pediatr Nurs. 1995;21(5):439–40; 43. 8684845

[2] Bahrami N Soleimani MA Chan YH Ghojazadeh M Mirmiran P. Menarche age in Iran: A meta-analysis. Iran J Nurs Midwifery Res. 2014;19(5):444–50. 25400670 PMC4223959

[3] Alavi M Poushaneh K Khosravi A. Puberty health: knowledge, attitude and practice of the adolescent girls in Tehran, Iran. 2009;8(1):59–65.

[4] Mosavi SA Babazadeh R Najmabadi KM Shariati M. Assessing Iranian adolescent girls’ needs for sexual and reproductive health information. J Adolesc Health. 2014;55(1):107–13. 24560307 10.1016/j.jadohealth.2013.11.029

[5] Chrisler JC. Teaching taboo topics: menstruation, menopause, and the psychology of women. Psychol Women Q. 2013;37(1):128–32.

[6] Costos D Ackerman R Paradis L. Recollections of menarche: Communication between mothers and daughters regarding menstruation. Sex Roles. 2002; 46(1–2):49–59.

[7] Dasgupta A Sarkar M. Menstrual hygiene: how hygienic is the adolescent girl? Indian J Community Med. 2008;33(2):77–80. 19967028 10.4103/0970-0218.40872 PMC2784630

[8] Delaney J Lupton MJ Toth E. The curse: A cultural history of menstruation. 1st ed Chicago: University of Illinois Press; 1988 283 p.

[9] Lawan UM Yusuf NM Musa AB. Menstruation and menstrual hygiene amongst adolescent school girls in Kano, northwestern Nigeria. Afr J Reprod Health. 2010;14(3):201–7. 21495614

[10] Tegegn A Yazachew M Gelaw Y. Reproductive health knowledge and attitude among adolescents: a community based study in Jimma Town, Southwest Ethiopia. Ethiop J Health Dev. 2016;22(3): 43–51.

[11] Statistical center of Iran April 2018. POPULATION BY AGE GROUPS ; [cited 2018 Jul 17]. Available from: https://www.amar.org.ir/english/Population-and-Housing-Censuses.

[12] Afghari A Eghtedari S Pashmi R Sadri GH. Effects of puberty health education on 10–14 year-old girls’ knowledge, attitude, and behavior. Iran J Nurs Midwifery Res. 2008;13(1):38–41.

[13] Rabiepoor S Valizadeh R Barjasteh S. Study of Menstrual Attitudes and Knowledge among Post-menarcheal Students, in Urmia, North West of Iran. Int J Pediatr. 2017;5(5):4991–5001.

[14] Alizadeh SH Zareban I Rakhshani F Shahraki Pour M Shamaian RN. The effects of education on knowledge attitudes and behavior of students of high schools in zahedan, 2011. 2013;12(2):113–23.

[15] Barati M Allahverdipour H Hidarnia A Niknami S Bashirian S. Belief-based tobacco smoking scale: evaluating the psychometric properties of the theory of Planned behavior’s constructs. Health Promot Perspect. 2015;5(1):59–71. 26000247 10.15171/hpp.2015.008 PMC4430699

[16] Bashirian S Hidarnia A Allahverdipour H Hajizadeh E. The theory-based substance abuse prevention program for adolescents. Health Educ Health Promot. 2012;1(1):3–12. 22888715

[17] DarabiFYaseriMKavehMHKhalajabadi FarahaniFMajlessiFShojaeizadehD. The effect of a theory of planned behavior-based educational intervention on sexual and reproductive health in Iranian adolescent girls: a randomized controlled trial. J Res Health Sci. 2017;17(4):e00400.29233954

[18] Darabi F Kaveh MH Majlessi F Farahani FKA Yaseri M Shojaeizadeh D. Effect of theory-based intervention to promote physical activity among adolescent girls: a randomized control trial. Electron Physician. 2017;9(4):4238–47. 28607661 10.19082/4238 PMC5459298

[19] Kaveh MH Darabi F Khalajabadi-Farahani F Yaseri M Kaveh MH Mohammadi MJ The impact of a tpb-based educational intervention on nutritional behaviors in iranian adolescent girls: a randomized controlled trial. Fresenius Environ Bull. 2018;27(6):4349–56.

[20] Cleland J Ali MM. Sexual abstinence, contraception, and condom use by young African women: a secondary analysis of survey data. Lancet. 2006; 368(9549):1788–93. 17113428 10.1016/S0140-6736(06)69738-9

[21] Lawshe CH. A quantitative approach to content validity. Pers Psychol. 1975;28(4):563–75.

[22] Waltz CF Bausell BR. Nursing Research: Design Statistics and Computer Analysis: Davis F A; 1981. 362 p.

[23] Mitzenmacher M Upfal E. Probability and computing: randomization and probabilistic techniques in algorithms and data analysis. 2nd ed UK: Cambridge university press; 2017 463 p.

[24] Westland JC. Lower bounds on sample size in structural equation modeling. Electron Commer Res Appl. 2010;9(6):476–87.

[25] Mouritsen A Johansen ML Wohlfahrt-Veje C Hagen CP Tinggaard J Mieritz MG Determination of adrenal volume by MRI in healthy children: associations with age, body size, pubertal stage and serum levels of adrenal androgens. Clin Endocrinol (Oxf). 2014;81(2):183–9. 24455980 10.1111/cen.12414

[26] Escandari N Alipour Z Lamyian M Ahmaritehrani H Hajizadeh E Mokhah S. Validity and reliability of the international AIDS questionnaire for Iranian student population. J Arak Univ Med Sci. 2013;15(10):1–12.

[27] Afghari A Eghtedari Sh Pashmi R Hossein Sadri G. Effects of puberty health education on 10–14 year-old girls’ knowledge, attitude, and behavior. Iran J Nurs Midwifery Res. 2008;13(1):38–41.

[28] Valizade R Taymoori P Yousefi FY Rahimi L Ghaderi N The effect of puberty health education based on health belief model on health behaviors and preventive among teen boys in Marivan, north west of Iran. Int J Pediatr. 4(8):3271–81.

[29] Bonett DG Wright TA. Cronbach’s alpha reliability: interval estimation, hypothesis testing, and sample size planning. J Organ Behav. 2015;36(1):3–15.

